# The Hologenome Concept: Helpful or Hollow?

**DOI:** 10.1371/journal.pbio.1002311

**Published:** 2015-12-04

**Authors:** Nancy A. Moran, Daniel B. Sloan

**Affiliations:** 1 Department of Integrative Biology, University of Texas at Austin, Austin, Texas, United States of America; 2 Department of Biology, Colorado State University, Fort Collins, Colorado, United States of America

## Abstract

With the increasing appreciation for the crucial roles that microbial symbionts play in the development and fitness of plant and animal hosts, there has been a recent push to interpret evolution through the lens of the “hologenome”—the collective genomic content of a host and its microbiome. But how symbionts evolve and, particularly, whether they undergo natural selection to benefit hosts are complex issues that are associated with several misconceptions about evolutionary processes in host-associated microbial communities. Microorganisms can have intimate, ancient, and/or mutualistic associations with hosts without having undergone natural selection to benefit hosts. Likewise, observing host-specific microbial community composition or greater community similarity among more closely related hosts does not imply that symbionts have coevolved with hosts, let alone that they have evolved for the benefit of the host. Although selection at the level of the symbiotic community, or hologenome, occurs in some cases, it should not be accepted as the null hypothesis for explaining features of host–symbiont associations.

## Abstract 2

It is accepted that microbial symbionts are important to the biology of their hosts, but this essay asks what evidence is needed to determine whether symbionts and hosts have coevolved and whether they form higher units of evolutionary selection.

The ubiquity and importance of microorganisms in the lives of plants and animals are ever more apparent, and increasingly investigated by biologists. Suddenly, we have the aspiration and tools to open up a new, complicated world, and we must confront the realization that almost everything about larger organisms has been shaped by their history of evolving from, then with, microorganisms [1]. This development represents a dramatic shift in perspective—arguably a revolution—in modern biology.

Do we need to revamp basic tenets of evolutionary theory to understand how hosts evolve with associated microorganisms? Some scientists have suggested that we do [2], and the recently introduced terms “holobiont” and “hologenome” encapsulate what has been described as an “emerging postmodern synthesis” [3]. Holobiont was initially used to refer to a host and a single inherited symbiont [4] but was later extended to a host and its community of associated microorganisms, specifically for the case of corals [5]. The idea of the holobiont is that a host and its associated microorganisms must be considered as an integrated unit in order to understand many biological and ecological features.

The later introduction of the term hologenome [2,6,7] sought to describe a holobiont by its genetic composition. The term has been used in different ways by different authors, but in most contexts a hologenome is considered a genetic unit that represents the combined genomes of a host and its associated microorganisms [8]. This non-controversial definition of hologenome is linked to the idea that this entity has a role in evolution. For example, Gordon et al. [1,9] state, "The genome of a holobiont, termed the hologenome, is the sum of the genomes of all constituents, all of which can evolve within that context." That last phrase is sufficiently general that it can be interpreted in any number of ways. Like physical conditions, associated organisms can be considered as part of the environment and thus can be sources of natural selection, affecting evolution in each lineage.

But a more sweeping and problematic proposal is given by originators of the term, which is that "the holobiont with its hologenome should be considered as the unit of natural selection in evolution" [2,7] or by others, that “an organism’s genetics and fitness are inclusive of its microbiome” [3,4]. The implication is that differential success of holobionts influences evolution of participating organisms, such that their observed features cannot be fully understood without considering selection at the holobiont level. Another formulation of this concept is the proposal that the evolution of host–microbe systems is “most easily understood by equating a gene in the nuclear genome to a microbe in the microbiome” [8]. Under this view, interactions between host and microbial genotypes should be considered as genetic epistasis (interactions among alleles at different loci in a genome) rather than as interactions between the host’s genotype and its environment.

While biologists would agree that microorganisms have important roles in host evolution, this statement is a far cry from the claim that they are fused with hosts to form the primary units of selection, or that hosts and microorganisms provide different portions of a unified genome. Broadly, the hologenome concept contends, first, that participating lineages within a holobiont affect each other’s evolution, and, second, that that the holobiont is a primary unit of selection. Our aim in this essay is to clarify what kinds of evidence are needed for each of these claims and to argue that neither should be assumed without evidence. We point out that some observations that superficially appear to support the concept of the hologenome have spawned confusion about real biological issues (Box 1).

**Box 1. Misconceptions Related to the Hologenome Concept**

**Misconception #1**: Similarities in microbiomes between related host species result from codiversification.

**Reality**: Related species tend to be similar in most traits. Because microbiome composition is a trait that involves living organisms, it is tempting to assume that these similarities reflect a shared evolutionary history of host and symbionts. This has been shown to be the case for some symbioses (e.g., ancient maternally inherited endosymbionts in insects). But for many interactions (e.g., gut microbiota), related hosts may have similar effects on community assembly without any history of codiversification between the host and individual microbial species (Fig 1B).

**Misconception #2**: Parallel phylogenies of host and symbiont, or intimacy of host and symbiont associations, reflect coevolution.

**Reality**: Coevolution is defined by a history of reciprocal selection between parties. While coevolution can generate parallel phylogenies or intimate associations, these can also result from many other mechanisms.

**Misconception #3:** Highly intimate associations of host and symbionts, involving exchange of cellular metabolites and specific patterns of colonization, result from a history of selection favoring mutualistic traits.

**Reality:** The adaptive basis of a specific trait is difficult to infer even when the trait involves a single lineage, and it is even more daunting when multiple lineages contribute. But complexity or intimacy of an interaction does not always imply a long history of coevolution nor does it imply that the nature of the interaction involves mutual benefit.

**Misconception #4**: The essential roles that microbial species/communities play in host development are adaptations resulting from selection on the symbionts to contribute to holobiont function.

**Reality**: Hosts may adapt to the reliable presence of symbionts in the same way that they adapt to abiotic components of the environment, and little or no selection on symbiont populations need be involved.

**Misconception #5**: Because of the extreme importance of symbionts in essential functions of their hosts, the integrated holobiont represents the primary unit of selection.

**Reality**: The strength of natural selection at different levels of biological organization is a central issue in evolutionary biology and the focus of much empirical and theoretical research. But insofar as there is a primary unit of selection common to diverse biological systems, it is unlikely to be at the level of the holobiont. In particular cases, evolutionary interests of host and symbionts can be sufficiently aligned such that the predominant effect of natural selection on genetic variation in each party is to increase the reproductive success of the holobiont. But in most host–symbiont relationships, contrasting modes of genetic transmission will decouple selection pressures.

**Fig 1:**
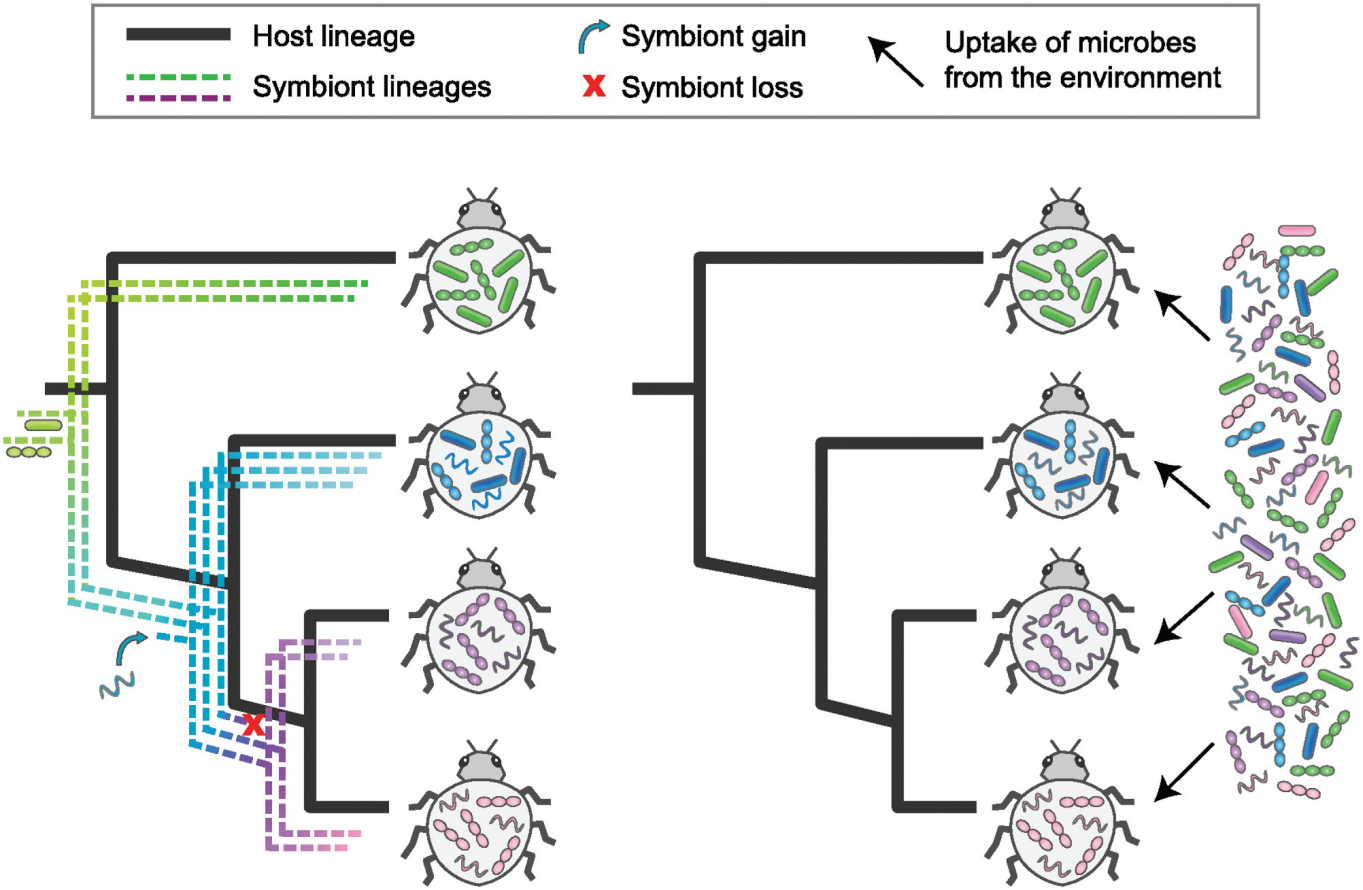
Alternative evolutionary processes can result in related host species harboring similar symbiont communities. Left panel: Individual symbiont lineages retain fidelity to evolving host lineages, through co-inheritance or other mechanisms, with some gain and loss of symbiont lineages over evolutionary time. Right panel: As host lineages evolve, they shift their selectivity of environmental microbes, which are not evolving in response and which may not even have been present during host diversification. In both cases, measures of community divergence will likely be smaller for more closely related hosts, but they reflect processes with very different implications for hologenome evolution. *Image credit*: *Nancy Moran and Kim Hammond*, *University of Texas at Austin*.

## Coevolution Versus Codiversification Versus Phylosymbiosis

Host associations with microorganisms often display several features that seem, at first glance, to support the view that they have evolved together as a unit. A particular host species often associates with a characteristic set of microbial species, and related hosts tend to have similar microbiota, suggesting the possibility of shared evolutionary history of the host and its microbial communities. Often, the relationships are intimate and highly specific, in the sense that microorganisms colonize specific tissues and exchange metabolites with hosts. What do such observations truly imply about the occurrence of host–microorganism coevolution or shared evolutionary history? By definition [10,11], coevolution requires that each lineage undergoes evolutionary change due to selective forces imposed by the other lineages. So how can we recognize when holobionts are shaped by the coevolution of participants, and furthermore whether this coevolution reflects selection at the level of the holobiont unit?

Coevolution is often assumed based on observing intimate associations between hosts and microorganisms, but intimacy of an association does not require a history of coevolution. In some cases, microorganisms colonize a host in an intimate and characteristic manner, and have a major impact on host fitness, yet are not evolving in response to the host. For example, *Pseudomonas aeruginosa* colonization of the human lung, associated with cystic fibrosis, impacts the host even though the lung is probably not a significant habitat for *P*. *aeruginosa*, which is primarily a soil-dwelling organism. Even relationships that bear striking similarities to highly coevolved mutualisms may not actually involve coevolution. For example, photosynthetic sea slugs are able to capture chloroplasts from specific algal food sources and maintain them for up to months at a time [12]. While it is tempting to imagine that the algae and their chloroplasts have somehow evolved to facilitate this spectacular example of a symbiotic interaction, this is entirely inconsistent with the current understanding of this relationship as an evolutionary dead-end for the algae.

Coevolution appears more likely when lineages have a shared evolutionary history, as evidenced by a pattern of codiversification (also called cospeciation or parallel phylogenesis) where symbiont and host lineages show matching phylogenetic trees (Fig 1A). By itself, however, codiversification does not imply coevolution. In fact, organisms with no interaction at all sometimes diversify in parallel if subjected to the same series of geographic isolation events [13]. Codiversification can also reflect unidirectional selection, in which a symbiont or parasite adaptively tracks changes in hosts but not vice versa. An example might be *Helicobacter pylori*, which has evolved in parallel with human populations [1,14]. Whether human populations have evolved in response is not evident. A strong pattern of codiversification—perfect matching of phylogenies of numerous species of host and associated symbiont—supports some type of shared history but does not demonstrate coevolution.

In fact, coevolution is hard to prove. This is illustrated by its past contentiousness among researchers of plant–animal interactions. Under one view, plants produce toxic molecules as metabolic side-products and specialized insect herbivores adapt to overcome these [2,15]. Alternatively, insects and plants have coevolved: toxins evolve as defensive adaptations and insects reciprocate with adaptations to neutralize these toxins. But strong evidence for coevolution is not impossible. In the case of insects and plants, coevolution has been strongly supported by studies showing sequential evolution of defenses and counter-defenses in butterflies and their food plants [3,16] and recent changes in toxin content in plant populations differing in herbivore exposure [4,17].

Sometimes coevolution or shared evolutionary history is presumed because particular host species associate with characteristic microbial species or communities. But this also does not imply that host and microorganisms have evolved in response to one another, or even that they have evolved in the presence of one another. For example, it has been demonstrated that showerheads are colonized by characteristic microbial communities that represent a highly selective subset of all water-borne microorganisms [5,18]. This is expected, as showerheads provide a distinctive habitat and resources, suitable for particular sets of organisms. But showerheads and microorganisms have not coevolved. Likewise, a host may be highly selective, permitting only a small subset of environmental organisms to colonize, without sharing any evolutionary history with them.

Often microbial communities are more similar among closely related hosts than among more distantly related ones [2,6,7,19–21]. “Phylosymbiosis” is a term coined recently for the idea that microbial communities change in a way that reflects host phylogeny [8,22]. But a relationship of host phylogeny to symbiont community composition can arise from two very different processes. First, ancestral associated microorganisms may diversify in parallel with the host, through continuous association of evolving lineages; i.e., they codiversify (Fig 1A). This is the case for organelles and eukaryotic cells, and for obligate symbionts and their host insects, such as *Buchnera* and aphids [23]. In this case, changes in microbiota during host evolution are due to fixation of mutations in evolving symbiont lineages, and the pattern supports long-term associations of symbionts with host lineages. Potentially, though not necessarily, this is linked to their acting on one another as reciprocal selective forces, i.e., coevolving. Alternatively, more related host species may simply be colonized by similar sets of microbial species from the environment (Fig 1B) [1,9,19,22]. In this case, changes in microbiota composition are due to differences in community assembly rather than genealogy. The microorganisms themselves need not have evolved at all, and need not have shared a long history with their hosts. In fact, this second pattern, that closely related host species share similar microbial communities, is expected under most evolutionary scenarios. Closely related species typically have greater resemblance than distantly related species for any phenotypic feature, be it genetic, behavioral, or ecological. Thus, this situation suggests nothing one way or the other about whether host and symbionts have coevolved or have a shared evolutionary history.

## Hologenomes As the Units of Selection

Although sometimes hard to demonstrate, coevolution and codiversification are likely common for hosts and associated microorganisms. But the proposals that the hologenome is the primary unit of selection and that microbes can be equated to genes within a larger genome are far more sweeping claims. They imply that selection on constituent genomes acts primarily to increase fitness at the level of the holobiont, that is, that conflicts between the evolutionary interests of host and symbionts are suppressed due to selection at the higher level. Even demonstrating coevolution does not necessarily imply that hologenomes are a significant unit of selection. In fact, most documented cases of interspecific coevolution involve antagonistic changes such as those of hosts and pathogens, or plants and herbivores.

A host’s survival depends on its interactions with microorganisms, and hosts are, therefore, under selection to encourage colonization by beneficial bacteria and to discourage colonization by harmful ones. However, such selection can often be interpreted in the simple context of a host adapting to the microorganisms in its environment, including those involved in antagonistic coevolution. For the hologenome to be the primary unit of selection, the evolution of microbial lineages must also be contributing to increased holobiont fitness. This view has many parallels with the idea of group selection: that natural selection acts on groups of individuals within a species, and not only on the individuals. Group selection can and does occur in some circumstances, but the required conditions are restrictive and often depend on groups that consist of genetic relatives [2,7,24] or the enforcement of a system of mutual policing to limit cheating [3,4,25]. Early and oversimplified views of group selection (e.g., that ungulate herds have evolved to avoid overexploiting rangeland plants, so as to conserve resources for future generations [8,26]) failed to recognize that population-level adaptations are unstable under most circumstances, due to susceptibility to invasion by selfish individuals (e.g., that eat more than their share and thereby reproduce more [10,11,27]). Many of the same issues arise for the proposal that hologenomes are the primary units of selection: what mechanisms are there to limit the tendency of microorganisms to evolve selfish traits at the expense of fitness of the holobiont?

Do hosts and their associated microorganisms ever form new units of selection, resulting in phenotypes only understandable as arising from selection on hologenomes? And, if so, when and how often? The answer to the first question is yes. Heritable obligate symbioses such as those of mitochondria and eukaryotes, or of insects and obligate bacterial symbionts that provision nutrients, provide clear cases in which fitness of each party is directly dependent on fitness of the other. Furthermore, this codependence is maintained, in some cases, for many millions of generations through the co-transmission of genomes of both parties. In the aphid–*Buchnera* symbiosis, the bacterium *Buchnera* provides nutrients to aphid hosts and is passed directly from mother to progeny. Selection acts on *Buchnera* to increase female fecundity and on the aphid to maintain the *Buchnera* upon which it depends. Both the aphid and the *Buchnera* have specific features that can only be interpreted as adaptations to promote the fitness of the holobiont: both parties cooperate in the production of a joint metabolism able to provide needed nutrients [2,6,7,12,28–30], and the aphid has evolved specialized mechanisms for housing and transmitting *Buchnera* [13,31]. In other insect groups, acquisitions of heritable bacteria and host adaptations for maintaining them appear to have been key adaptations in shifts between dietary niches, such as xylem versus phloem sap of plants [32,33]. Clearly, these systems are shaped by holobiont-level selection, acting both on gains and losses of heritable symbionts and on mutational changes in symbiont and host genomes [8].

But even in these extreme cases of co-inherited genomes, selection at the level of the hologenome fails to explain many features, which are understandable only by considering the distinct transmission routes and selective milieu of each partner. For mitochondria, the most intimate and coevolved example of symbiosis, traits sometimes oppose fitness interests of the “host,” resulting in cytonuclear conflict [34–36]. Selection acts at multiple levels simultaneously, and heritable symbionts can evolve as both helpers and antagonists. For example, maternally inherited symbionts such as *Wolbachia* and *Spiroplasma* provide insect hosts with protection against natural enemies [37,38] but also inflict costs on hosts by manipulating their reproductive systems to favor female progeny [39,40].

Thus, even when symbionts are vertically inherited and more readily regarded as part of the host genetics than as part of the environment, conflicts are rampant. To state that the hologenome is the primary unit of selection oversimplifies, and ignores a large body of theoretical and experimental work devoted to teasing apart how selection acts on these composite entities.

But the majority of microbial associations of multicellular animals and plants are non-heritable. Sometimes these too appear to involve selection on hologenomes. In the bobtail squid–*Vibrio fischeri* symbiosis, hosts are colonized repeatedly during their lives and employ specific mechanisms for recognizing and admitting their symbionts [41], and symbionts retain features (luminescence) that benefit hosts. These cases are intriguing, as their stability suggests that hosts have evolved effective policing mechanisms that prevent “selfish” symbionts from invading and spreading.

Gut-dwelling organisms are of particular interest, due to their potential to affect the health of animal hosts. To what extent are they selected at the hologenome level? The fidelity to a host and transmission routes vary enormously among host species and among gut symbionts. At one extreme, gut symbionts are directly transferred from mothers to progeny through mechanisms such as the deposition of a symbiont mass that is ingested by newly hatched progeny (e.g., [42]). Alternatively, maternal behaviors or gregarious lifestyles may effect direct transfer of gut organisms between members of a host species. But in many cases, gut organisms are promiscuous: they are picked up from the environment or from unrelated host individuals every generation, or repeatedly during the lives of single individuals as they develop. In some cases, gut communities appear to consist of microbial species that are widespread in the environment, so natural selection associated with the host gut may be inconsequential for these organisms. In many insects, gut communities are a highly selected subset of microbial species ingested with food but nonetheless are variable among host individuals and habitats [43–46].

Across this spectrum, how can we determine if and when selection at the hologenome level has an important evolutionary impact? Even in the highly dynamic cases, fitness interests of host and symbionts can align: symbiont fitness may depend on host longevity and growth rate, which may correlate with opportunities for symbiont replication and dispersal. Thus, opportunity for selection at the hologenome level exists, but it may be small or insignificant relative to selection on individual interacting genomes.

Reciprocal fitness benefits, i.e., mutualism, or developmental dependence of the host on its symbionts are sometimes taken as evidence of selection at the hologenome level. But even if microorganisms are effectively parasitic and provide no initial benefit to hosts, host development can evolve to become dependent on them. Thus, the striking finding that normal development of the vertebrate gut is dependent on microorganisms [47] does not imply that gut inhabitants have evolved to promote gut development, only that vertebrates have evolved dependence on a part of the environment that is reliably present. Just as day length is used by many plants as a cue for initiating flowering times, presence of particular organisms can provide useful developmental cues. Another example is the role of bacteria in promoting multicellularity in choanoflagellates [48]: this finding does not imply that the bacteria evolved to carry out this role, only that they have been adopted by choanoflagellates as a developmental cue.

## What Is the Null Hypothesis?

Many features of eukaryotes, including ourselves, cannot be understood without taking into account microbial associates, which are themselves evolving. Clearly we need to study organisms in the context of these associations, but how should we formulate our questions and hypotheses? We argue that the *wrong* approach is to start with the assumption that associated organisms have evolved to function as a cooperative unit and that the task is simply to characterize mutually beneficial adaptations. This assumption is often incorrect, and embracing it uncritically will slow progress. A more parsimonious approach is to adopt the null hypothesis that interacting lineages have not evolved exceptional hologenome-selected traits, and to test specific hypotheses regarding such traits.

For many purposes, the issue of whether organisms have evolved due to selection on a hologenome is irrelevant. For the purpose of understanding how symbionts affect human health or productivity of agricultural crops, one may simply want to know the current effect of a symbiont on the host, whatever the evolutionary history may have been. But careful wording that does not invoke dubious evolutionary mechanisms is still important. Sometimes hosts and symbionts have coadapted for mutual benefit, but even in these cases, an element of antagonism persists due to the differences in fitness interests of the interacting genomes. In other cases, hosts have evolved to be dependent on microorganisms, even though the latter have not evolved in response. The central claim of the hologenome concept, that a host and its microbiome together form the primary unit of selection, is sometimes true, and sometimes false; its validity will depend on the particular case. In answer to the question posed in the title, we suggest that the varied and often inconsistent interpretations of the hologenome concept have been a source of more confusion than clarity in understanding the evolution of host–microbe interactions. Advances will come from empirical studies that start with rigorous assumptions and a clear framework for detecting coevolution and teasing apart levels of selection.
